# Fluorescence-based dual detection of chlortetracycline and Pb^2+^ using nitrogen/phosphorus co-doped carbon quantum dots

**DOI:** 10.1039/d5ra05918f

**Published:** 2025-11-05

**Authors:** S. Viji, Srimathi Priya L., A. Dinesh, Radhakrishnan K., Manikandan Ayyar, Prabhu Paramasivam, Gnanasekaran Lalitha, V. Mohanavel, M. Santhamoorthy, G. Ramachandran, S. Santhoshkumar, Ankush Mehta, N. Subasree

**Affiliations:** a Department of Chemistry, Dr Ambedkar Govt. Arts College (Autonomous), Affiliated to the University of Madras Vyasarpadi Chennai – 600 039 Tamil Nadu India puppyviji82@gmail.com ramuvec@gmail.com; b Department of Natural Resource Management, Horticultural College and Research Institute (HC & RI), Tamil Nadu Agricultural University (TNAU) Periyakulam – 625 604 Tamil Nadu India srimathipriya.agri@gmail.com; c Department of Chemistry, K. Ramakrishnan College of Engineering (Autonomous), Affiliated to the Anna University Samayapuram Trichy – 621112 Tamil Nadu India dineshphdchem@gmail.com; d Department of Chemistry, Karpagam Academy of Higher Education Coimbatore Tamil Nadu – 641021 India raddhakrishnan21@gmail.com; e Centre for Material Chemistry, Karpagam Academy of Higher Education Coimbatore Tamil Nadu – 641021 India manikandan.frsc@gmail.com; f Centre for Research Impact & Outcome, Chitkara University Institute of Engineering and Technology, Chitkara University Rajpura 140401 Punjab India; g Instituto de Alta Investigación, Universidad de Tarapacá Arica – 1000000 Chile lalitha1887@gmail.com; h Centre for Sustainable Materials Research, Department of Mechanical Engineering, Academy of Maritime Education and Training (AMET), Deemed to be University Kanathur Chennai 603112 Tamil Nadu India mohanavel2k18@gmail.com; i School of Chemical Engineering, Yeungnam University Gyeongsan 38541 Republic of Korea santham83@gmail.com; j Department of Biochemistry, Saveetha Medical College and Hospital, Saveetha Institute of Medical and Technical Sciences Chennai Tamil Nadu India santhoshkumars.simats@gmail.com; k Marwadi University Research Center, Department of Mechanical Engineering, Faculty of Engineering & Technology, Marwadi University Rajkot 360003 Gujarat India ankush.mehta38@gmail.com; l Department of Chemistry, School of Basic Science, Vels Institute of Science, Technology and Advanced Studies (VISTAS) Pallavaram Chennai Tamil Nadu 600117 India suba25692@gmail.com; m Department of Mechanical Engineering, Mattu University Mettu – 318 Ethiopia drprabhu@meu.edu.et

## Abstract

A dual-mode fluorescence sensor composed of nitrogen/phosphorus co-doped carbon quantum dots (N/P-CQDs) was synthesized to detect chlortetracycline (CTC) and Pb^2+^ ions sequentially and selectively. The N/P-CQDs were synthesized through a green hydrothermal process using pomegranate peel juice as a renewable carbon source. The resulting N/P-CQDs exhibit blue emission with excitation and emission peaks at 400 and 475 nm, respectively, and a mean size of 7.5 nm. Chlortetracycline (CTC) quenches the fluorescence of N/P-CQDs *via* the inner filter effect (IFE), and then, it is recovered in the presence of Pb^2+^ ions, which proves the reversible on–off–on sensing mechanism of this sensor. The emission intensity exhibited a linear response to CTC (0–100 μM and LOD of 30 nM), while Pb^2+^ (5–100 μM) successfully restored the signal with an LOD of 18 μM. Furthermore, this sensor has excellent selectivity, stability and reproducibility at the optimized pH, ionic strength, and temperature. Its recovery values were about 92–98% in the real sample analysis of tomato, milk and river water, indicating that the N/P-CQD probe is applicable in the area of environmental and food safety monitoring.

## Abstract 2

Dual fluorescence detection of CTC and Pb^2+^ using N,P-co-doped carbon quantum dots.

## Graphical abstract

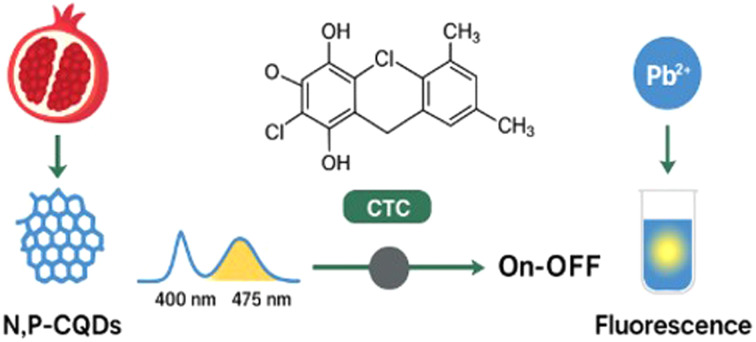

## 1. Introduction

Toxic pollutants such as lead and chlortetracycline act as double threats to human beings and the ecological balance, requiring quick responses and better monitoring techniques.^1^ Lead (Pb^2+^) is a toxic heavy metal that can cause neurodevelopmental abnormalities, renal impairment, and hematological issues, particularly in children.^2^ The extensive application of chlortetracycline (CTC) in veterinary and agricultural operations leads to its accumulation in ecosystems and the emergence of antimicrobial resistance.^3,4^ These contaminants endure in environmental matrices and food chains, ultimately jeopardizing the integrity of the ecosystem and human health.^5–7^ Therefore, the fabrication of sensitive, selective, and environmentally friendly sensing platforms capable of their simultaneous detection is crucial for safeguarding the environment and public health.

Modern scientific research has produced different state-of-the-art testing techniques for the analysis of CTC using electrochemistry, electrophoresis, microbiological methods, and HPLC systems.^8,9^ However, these methods require significant work by operators and costly equipment to perform effectively. On the other hand, CQD-based fluorescence sensors offer a quick, portable, and inexpensive alternative with superior sensitivity and selectivity. Additionally, heteroatom doping improves the optical characteristics of CQDs, allowing them to perform well even under real sample conditions in environmental and food monitoring. Recent research indicates that fluorescence spectroscopy represents a valuable tetracycline detection method when using sensitive quantum dot and noble metal nanocluster probes. The functionalization of CQDs with heteroatoms can improve their performance for the detection of tetracycline.^10^

Chromone and rhodamine-based fluorescent receptor materials provide exceptional sensitivity and selectivity for Cu^2+^ and Ce^2+^ ions.^11,12^ Nanomaterials are essential for the accurate design and optimization of sophisticated biosensing systems due to their unique recognition phenomena at the nanoscale. Among them, semiconductor nanoparticles (TiO_2_, ZnO, and CdS) improve electrochemical sensing by effective charge transfer, whereas noble metal nanoparticles (Au and Ag) allow visual detection through localized surface plasmon resonance (LSPR). Analyte separation and enrichment are made easier by magnetic nanoparticles (FeO_4_), and hybrid or bimetallic systems combine optical, electrical, and catalytic capabilities to provide greater stability and selectivity.^13^ These adaptable nanostructures serve as the basis for combining MOFs/COFs with 2D materials to create sensing platforms that are incredibly durable, selective, and efficient.

The exceptional properties of carbon-based nanomaterials, such as their non-toxic nature, biocompatibility, robust mechanical strength, and thermal stability, have garnered significant research interest, positioning them as promising candidates for a wide range of applications in health, environmental monitoring, and advanced technological fields. Among them, carbon quantum dots (CQDs) are zero-dimensional (0D) structures with sizes typically less than 10 nm. Their surface functionalization makes them effective to detect various chemical species, with a strong focus on environmental pollutant monitoring and chemical sensing.^14,15^ The doping of CQDs with N, P, S and B atoms leads to enhanced fluorescence through changes in their electronic structure and electron density. The doping process enhances the performance of CQDs through higher quantum yield detection, while increasing their ability to identify different environmental pollutants including metal ions, pesticides, pharmaceuticals and phenolic compounds.^16,17^ However, the methods for the synthesis of doped CQDs still poses challenge for large-scale production and real-world applications, highlighting the need for more cost-effective and simplified synthesis routes.^18^

The special features of CQDs including brightness under UV light and easy dispersal in water make them perfect for creating sensors that work well in both laboratory and biological applications.^19,20^ Scientists have found that nitrogen-doped carbon quantum dots show good results but encounter problems when exposed to metal ions and lose their efficiency, while co-doping with nitrogen and phosphorus works better to solve these problems.^21,22^ Also, the stability and photoluminescent behaviour of these materials can be improved by using phosphoric acid and mixed aliphatic amines or special dopants. Carbon dots with nitrogen and phosphorus co-doping showed a good performance in the medical detection of chromium(vi) and iron (Fe^3+^) ions at very low levels. Additionally, the combination of sulfur, nitrogen and phosphorus heteroatoms with carbon quantum dots leads to improved fluorescence, as demonstrated in current studies.^23^

**Scheme 1:**
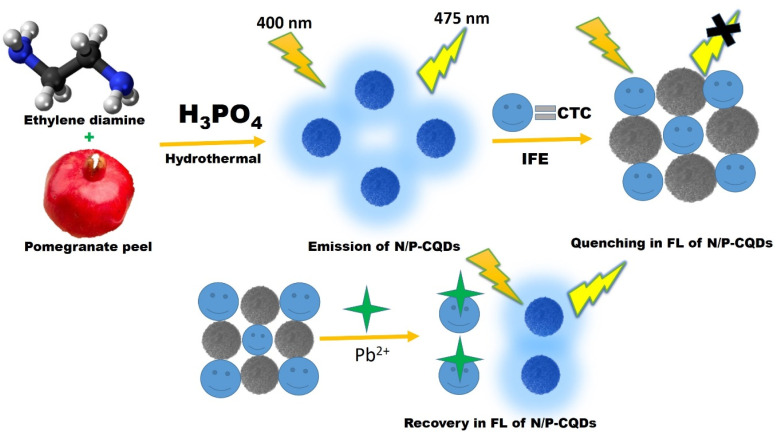
Schematic of the principle for the sensitive detection of CTC using N/P-CQDs.

The present study developed a rapid, single-step hydrothermal method for the synthesis of nitrogen/phosphorus co-doped carbon quantum dots (N/P-CQDs) directly using pomegranate peel juice, a biomass waste material, as a carbon source and naturally occurring reducing/stabilizing agent. This method offers a cost-effective, sustainable, and green alternative to the traditional multi-step CQD synthesis. The N/P-CQDs are a one-probe, two-analyte fluorescence sensor that can sensitize chlortetracycline (CTC) and Pb^2+^ ions through a distinct on–off–on response mechanism, requiring no complicated surface modifications. The CQD sensor employs an integrated sensing architecture and cost-effective precursors, enabling the simultaneous detection of multiple analytes with high sensitivity and selectivity. Moreover, pomegranate peel juice can be used to easily synthesize highly fluorescent N/P-CQDs, which integrates green synthesis with the sensing of multiple environmentally contaminants, thereby offering an entirely new and viable platform for multifunctional multi-sensing applications (Scheme 1).

## 2. Materials and methods

### 2.1. Chemicals and reagents

The reagents and chemicals used in this study were purchased from reliable vendors. Sisco Research Laboratories Pvt. supplied the following reagents: phosphoric acid, ethylenediamine, potassium nitrate, sodium nitrate, sodium phosphate, sodium chloride, reduced glutathione, and aspartic acid. Alfa Aesar and Merck provided the antibiotics, which included streptomycin sulfate (STC), amoxicillin (AMP), enrofloxacin, ciprofloxacin, chloramphenicol (CLP), kanamycin sulfate (KMS), chlortetracycline (CTC), tetracycline (TC), and oxytetracycline (OTC), and copper(ii) nitrate, nickel(ii) nitrate, mercury(ii) nitrate, cobalt(ii) nitrate, lead(ii) nitrate, and iron(ii) nitrate. Before each experiment, ultrapure water from a Millipore water purification system, which has a resistance of 18.2 MΩ cm^−1^, was used to carefully create freshly made aqueous antibiotic solutions. All chemicals used were of analytical purity and used without further purification unless otherwise specified.

### 2.2. Characterization

Different instruments were used to characterize the structural and morphological properties of N/P-CQDs. The samples were examined by X-ray diffraction (XRD, Bruker D8 Advance, Cu K-alpha, 1.5406 Å). To identify the size of the particles and the lattice structure, transmission electron microscopy (TEM, JEOL JEM-2100) was used. To determine the functional groups, Fourier-transform infrared (FTIR) spectra were obtained using a PerkinElmer Spectrum Two FTIR spectrometer. To analyze the optical and emission properties, a UV-2600 spectrophotometer and photoluminescence (PL) spectrophotometer were used to obtain UV-Visible absorption spectra and photoluminescence (PL) spectra, respectively.

### 2.3. Synthesis of N/P co-doped CQDs

Pomegranate peel juice was utilized as a sustainable carbon precursor owing to its rich content of polyphenols, flavonoids, organic acids, and carbohydrates, which act as natural reducing and stabilizing agents during carbonization and surface passivation. The N/P-CQDs were synthesized *via* a hydrothermal route in a phosphoric acid-assisted medium. Briefly, 1000 μL of ethylenediamine was dissolved in 20 mL of ultrapure water, followed by the addition of 5 mL of freshly prepared pomegranate peel juice. The resulting mixture was ultrasonicated for 20 min to ensure complete homogenization. Subsequently, 2.5 mL of 10% phosphoric acid was added dropwise under continuous stirring. The homogeneous solution was then transferred to a Teflon-lined stainless-steel autoclave and subjected to hydrothermal treatment at 180 °C for 12 h. After natural cooling to room temperature, the product was purified through a syringe filter. The purified N/P-CQDs were concentrated under reduced pressure using a rotary evaporator and redispersed in ethanol. The final product was stored at 4 °C for subsequent applications.

### 2.4. Fluorescence-based CTC sensing

The fluorescence detection approach for chlortetracycline (CTC) and lead (Pb^2+^) required the establishment of a sensing system that uses N/P-CQDs and Tris–HCl buffer. The experimental methodology and the conditions are outlined as follows: a mixture of Tris–HCl buffer (10 mM, pH 8.0) and N/P-CQDs (0.1 mL) was combined to provide a sensing solution with a total volume of 1.5 mL. To evaluate the efficacy of the sensing solution, it was combined with different concentrations of CTC solutions in the range of 0–300 μM. The solution was precisely combined and thereafter incubated for 3 min at ambient temperature. Fluorescence spectra were obtained by acquiring emission spectra in the range of 400–700 nm at an excitation wavelength (*λ*_ex_) of 400 nm. The precise detection and quantification of CTC content were facilitated by variations in the relative fluorescence intensity (*F*_0_/*F*) of N/P-CQDs. Similarly, the recovery of the fluorescence of N/P-CQDs was observed by varying the concentration of Pb^2+^ (5–80 μM) and incubation for 3 min. The undoped CQDs exhibited a significantly lower quantum yield and diminished fluorescence response to CTC and Pb^2+^, underscoring the critical role of heteroatom doping in enhancing the optical properties and detection sensitivity.

### 2.5. Selectivity for ions and antibiotics

To assess their selectivity and specificity, a number of substances that can potentially impair the functionality of N/P-CQDs were exposed to the identical laboratory settings used for CTC (80 μM). The interference molecules such as enrofloxacin (EF), ciprofloxacin (CF), enrofloxacin (OC), TC, STC, AMP, KMS, CLP, anions, Cu^2+^, Ni^2+^, Hg^2+^ and Co^2+^ were maintained at a constant level of 100 μM. This experimental setup enables the sensitive detection of CTC using N/P-CQDs. The fluorescence studies were performed in triplicate, with error bars representing mean ± standard deviation (*n* = 3). In the experiments for the optimization of the parameters such as pH dependence, thermostability, and saline environment stability, the fluorescence intensity was measured at the emission maximum (*λ*_em_ = 475 nm) and excitation wavelength (*λ*_ex_ = 400 nm) because of its distinctive photoluminescence maximum, resulting in excellent sensitivity and reproducibility.

### 2.6. Chlortetracycline detection in real samples

The applicability of N/P-CQDs for the detection of chlortetracycline (CTC) and Pb^2+^ ions was evaluated using milk, tomato juice, and river water samples. All the samples were centrifuged and filtered using a 0.22 μm membrane before the analysis to eliminate suspended impurities. The milk samples were deproteinized with acetone nitrile, centrifuged, and then filtered to remove macromolecular interferents including proteins and fats in the sample. The samples of tomato juice and river water were accordingly diluted using deionized water. A recovery and detection test was then performed on each sample by spiking the samples with three concentrations of CTC (20, 30 and 40 μM). Sensitive detection and quantification were done using fluorescence spectroscopy, which revealed the high selectivity and stability of the N/P-CQDs in complex sample matrices.

### 2.7. Quantum yield determination

Quantum yield (QY) determination is crucial in evaluating the efficiency of the synthesized N/P-CQDs (carbon quantum dots). In this study, the reference standard of quinine sulfate in 0.1 M H_2_SO_4_ was employed to analyze the quantum yield. The N/P-CQDs showed a strong emission intensity at 475 nm (*λ*_ex_ 400 nm) with a quantum yield of 18.5%, demonstrating their high efficiency as a fluorescent probe.

The quantum yield (*Q*) was determined using the following equation:

1 *Q*_x_ = *Q*_std_(*I*_X_/*I*_std_)(*η*_X_^2^/*η*_std_^2^)(*A*_X_/*A*_std_)

where *Q*_x_ and *Q*_std_ stand for the quantum yields of synthesized N/P-CQDs and quinine sulfate, respectively. Water and H_2_SO_4_ have refractive indices of 2*η*_x_^2^ and 2*η*_std_^2^, respectively. The integrated emission areas of quinine sulfate and manufactured carbon quantum dots are represented by *I*_std_ and *I*_x_, respectively. The optical densities of quinine sulfate and produced carbon quantum dots are represented by *A*_std_ and *A*_x_, respectively. The absorbance of N/P-CQDs was kept below 0.1 at the excitation wavelength by the researchers to reduce the inner filter effect. During the experiment, particular experimental parameters were carefully set, such as the excitation and emission slit widths of 1 nm and 2 nm, respectively.

## 3. Results and discussion

### 3.1. Characterization of the N/P-CQDs

**Fig. 1:**
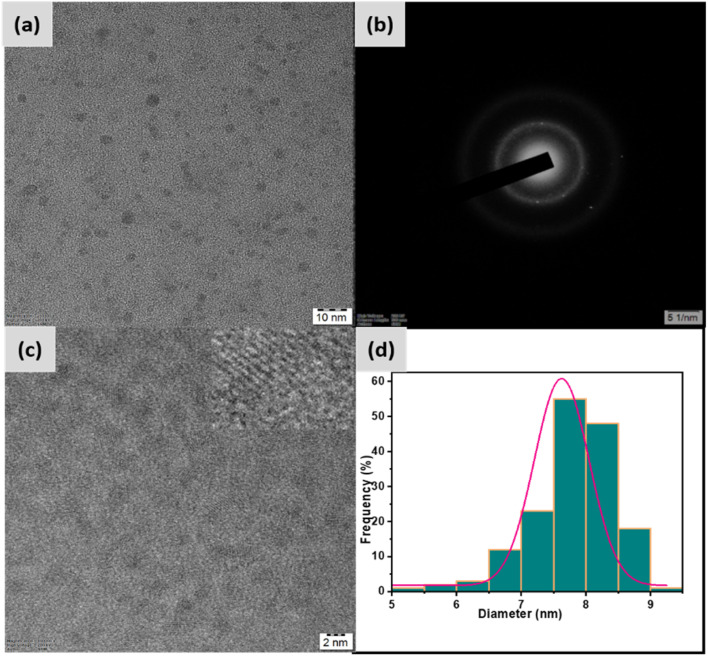
TEM analysis of the synthesized nanomaterial: (a) TEM image showing uniform spherical particles, (b) SAED pattern confirming its polycrystallinity, (c) HRTEM image revealing lattice fringes, and (d) particle size distribution.

The synthesized N/P-CQDs were comprehensively characterized using TEM, XPS, XRD, FTIR, and UV-Vis analyses. The N/P-CQDs showed strong fluorescence at 440 nm (*λ*_ex_ 360 nm) with a quantum yield of 18.5%, demonstrating their high efficiency as a fluorescent probe. TEM analysis was performed to identify the structural morphology of N/P-CQDs. Fig. 1a demonstrates the TEM images of the N/P-CQDs, depicting their morphology and size distribution. As shown in Fig. 1b, their SAED pattern exhibits distinct concentric rings, which is evidence that the sample is polycrystalline in nature. The high-resolution transmission electron microscopy (HRTEM) image reveals lattice fringes, which indicate strong crystallinity and a specified interplanar spacing (Fig. 1c). The histogram of the particle size distribution obtained from the transmission electron microscopy images displays a restricted size range and an average particle size of around 7.5 nm.

**Fig. 2:**
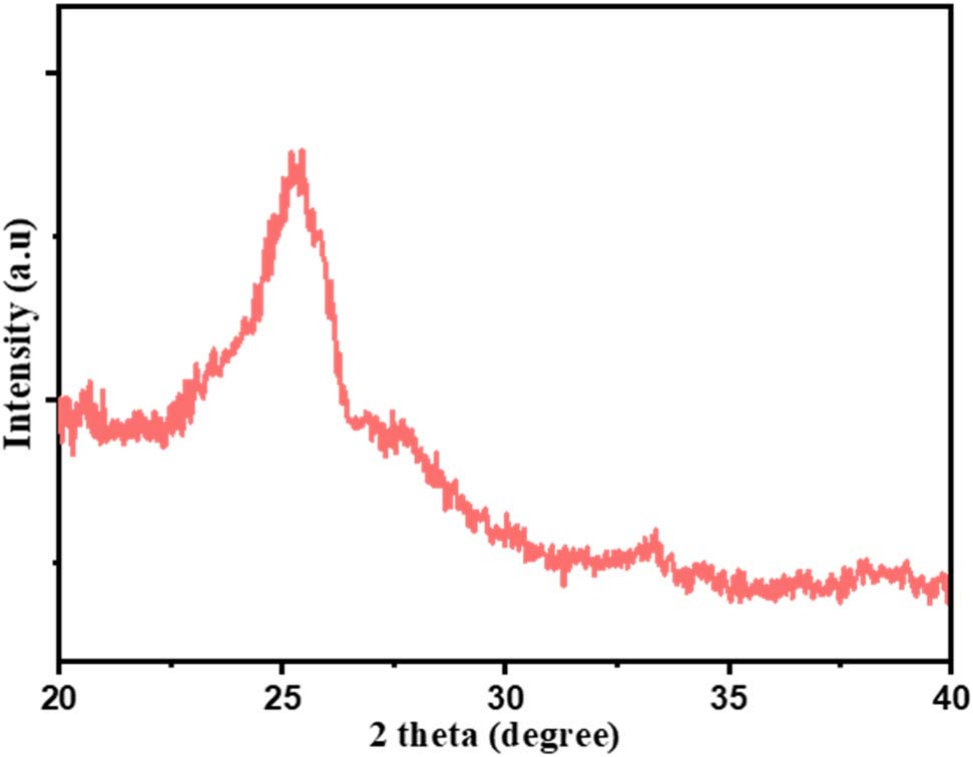
X-ray diffraction (XRD) pattern of carbon quantum dots (N/P-CQDs).

The X-ray diffraction (XRD) pattern of N/P-CQDs was analyzed to assess their crystalline structure, exhibiting a strong diffraction peak centered at 2*θ* = 25.3°. This peak corresponds to the (002) lattice plane within graphitic carbon, as depicted in Fig. 2. The presence of stacked aromatic domains or turbostratic carbon layers within the CQD structure is indicated by the presence of this peak. The broad width of the peak represents the possible formation of nanocrystalline graphitic domains. These domains are typical of amorphous carbon systems that have limited long-range organization. The discovered crystallinity is a factor that contributes to the stability of the N/P-CQDs as well as their improved photoluminescence.

**Fig. 3:**
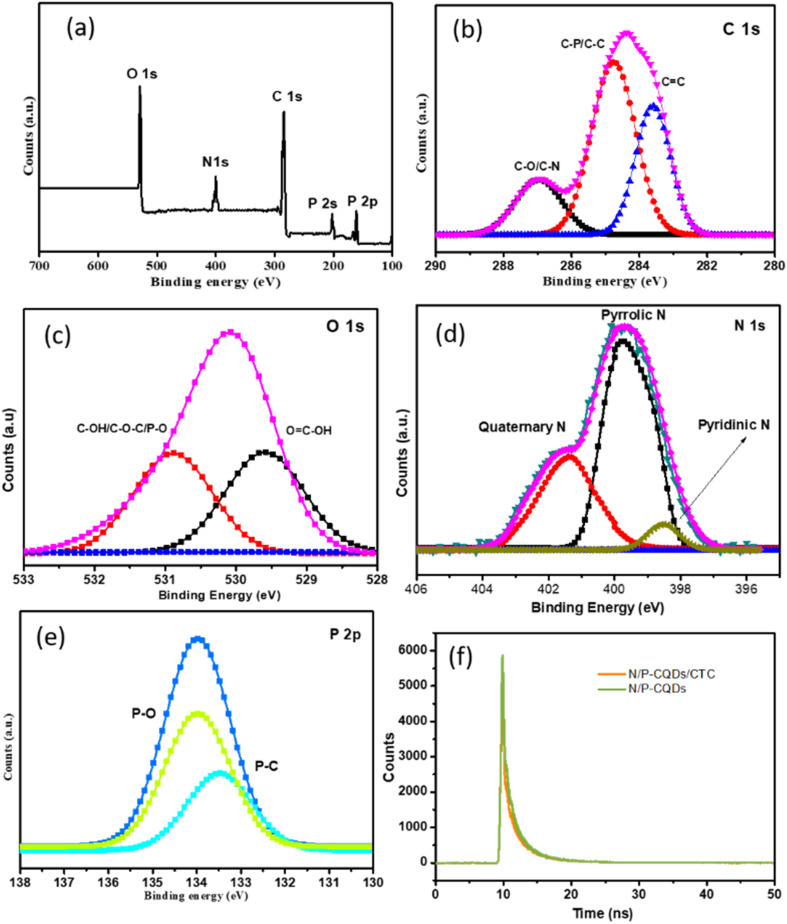
XPS analysis of carbon quantum dots (N/P-CQDs). (a) Full scan spectrum revealing overall elemental composition. High-resolution spectra focused on specific elements: (b) carbon, (c) oxygen, (d) nitrogen and (e) phosphorus, providing detailed insights into the chemical states and concentrations of each constituent in the synthesized N/P-CQDs. (f) Emission lifetime decay profiles of N/P-CQDs and with CTC.

X-ray photoelectron spectroscopy (XPS) analyses were performed to determine the surface functional groups and elemental composition responsible for the fluorescence and sensing performance of the probe. Fig. 3a presents the XPS spectrum of N/P-CQDs, which reveals the presence of components such as oxygen (O), carbon (C), nitrogen (N), and phosphorus (P). Doping the carbon quantum dots with nitrogen and phosphorus is demonstrated by this spectrum, which substantiates the previous results. Fig. 3b displays the results of the high-resolution peak fitting analysis of the C 1s spectra. This analysis reveals peaks corresponding to C=C bonding states (283.4 eV), C–OH bonding states (285.9 eV), and C–C/C–P bonding (284.6 eV). These peaks indicate that carbon and phosphorus interact with through bonding. The peaks in the O 1s spectra correspond to the bonding states of O–C–O and C–OH/C–O–C/P–O, located at the binding energies of 529.5 and 530.8 eV, respectively, as shown in Fig. 3c below. Graphitic nitrogen, pyridinic-like nitrogen, and pyrrolic-like nitrogen are the three principal peaks that can be detected in the N 1s spectrum (Fig. 3d). Graphitic nitrogen is the most characteristic of the three. 398.3, 399.7, and 401.3 eV are the respective energies at which these peaks can be found. These peaks correspond to different nitrogen states. The P 2p spectra indicate the presence of P–C and P–O bonds, as can be seen in Fig. 3e, corresponding to the peaks at 133.3 eV and 133.9 eV, respectively. These heteroatoms are recognised for their ability to modify the electronic structure by creating localised states and changing the electron density distribution within the carbon framework. Nitrogen, possessing a lone pair of electrons, can transfer electron density to the π-system of the carbon lattice, hence augmenting electron delocalisation and enhancing the quantum yield. Phosphorus, owing to its diminished electronegativity and capacity to create structural defects, can further improve the surface reactivity.^24,25^ These findings provide insights into the elemental composition of the material as well as the bonding interactions that occur within it.

**Fig. 4:**
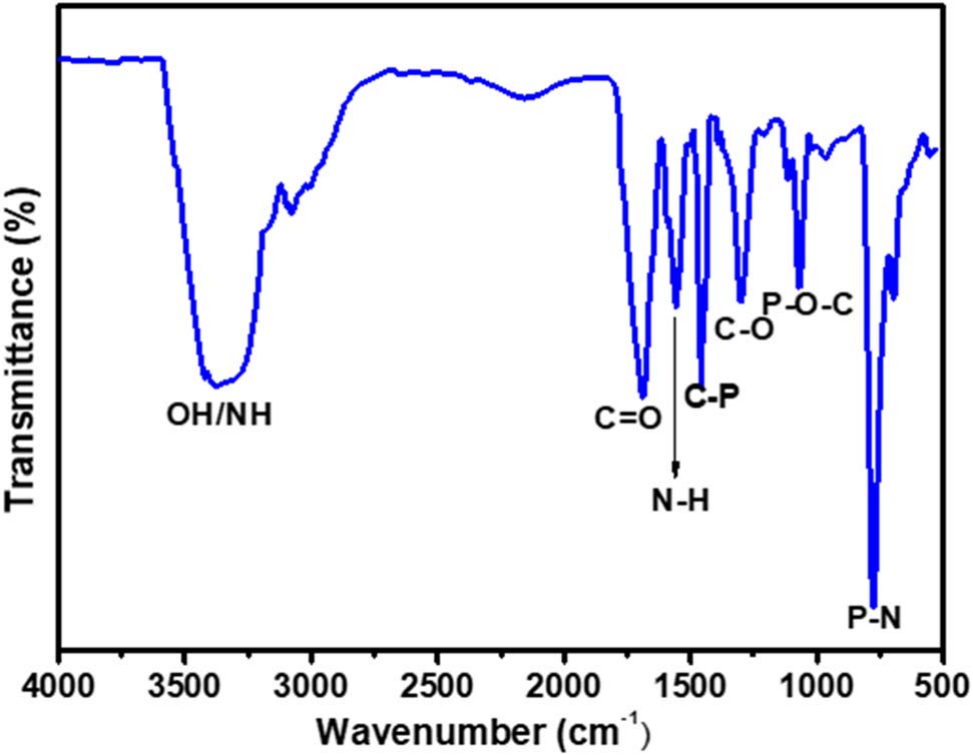
FTIR spectrum of N/P-CQDs.

Fourier transform infrared (FTIR) analysis was performed to determine the surface functional groups in the N/P-CQDs, as seen in Fig. 4. The stretching vibration of the OH/N–H bond was found to be responsible for the absorption peak located at 3354 cm^−1^, as determined by the researchers. The vibration of the C=O bond and the N–H bond in the amide group led to the appearance of two strong absorption bands. These bands matched the vibration of the amide group, which were located between 1708 and 1608 cm^−1^. In the process of determining the presence of C–O bond stretching vibration, it was established that it corresponded to the peak observed at 1294 cm^−1^, providing evidence for its existence. Among the various explanations for the peaks located at 1452, 1076, and 794 cm^−1^, they were identified to correspond to C–P, P–O–C, and P–N bonds, respectively. This provides evidence that phosphorus is chemically related to N/P-CQDs, demonstrating the present of this linkage.^26,27^ An essential understanding of the surface functionalization of N/P-CQDs can be gained from the numerous spectral patterns found in their infrared spectrum.

**Fig. 5:**
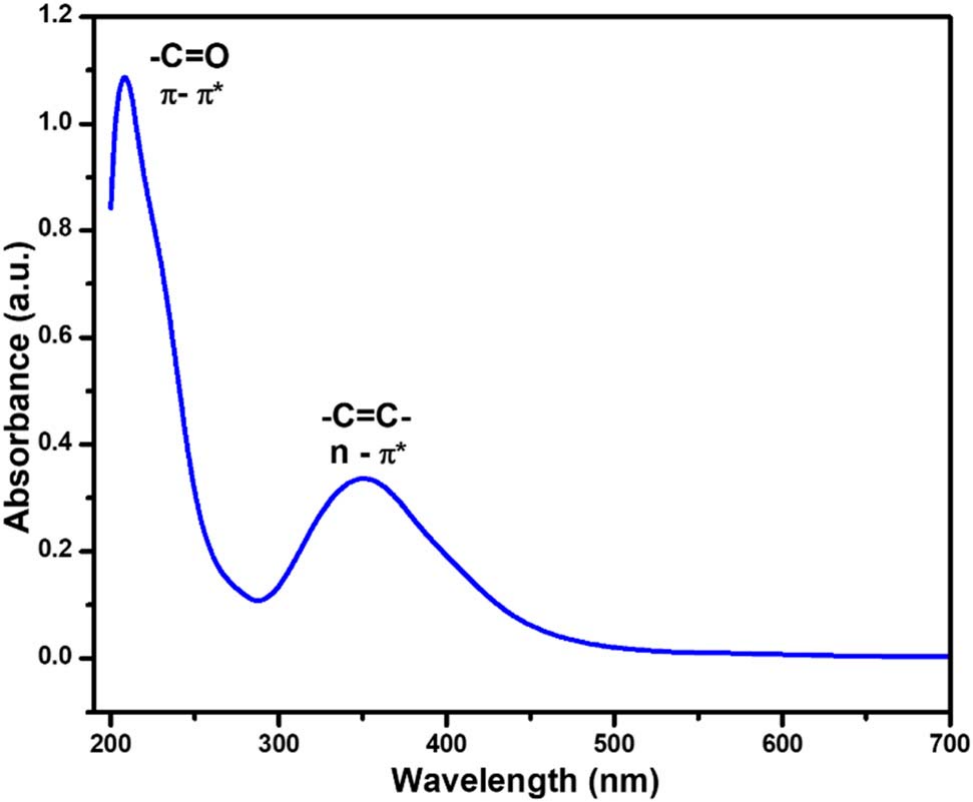
UV-vis absorbance spectrum of N/P-CQDs.

UV-Vis spectroscopy was employed to evaluate the optical properties of the synthesized N/P-CQDs. The N/P co-doped carbon quantum dots (N/P-CQDs) were observed to exhibit strong blue fluorescence under UV light at 365 nm. Their UV-vis absorption spectrum in the range of 200–700 nm had two strong absorption bands at about 220 nm and 365 nm, indicating Π–Π* and n–Π*, respectively, as shown in Fig. 5. These spectral properties of the heteroatom-doped CQDs once again confirm the successful inclusion of nitrogen and phosphorus in the carbon quantum dot-based structure.^28^

### 3.2. Optimization analysis of N/P-CQDs

**Fig. 6:**
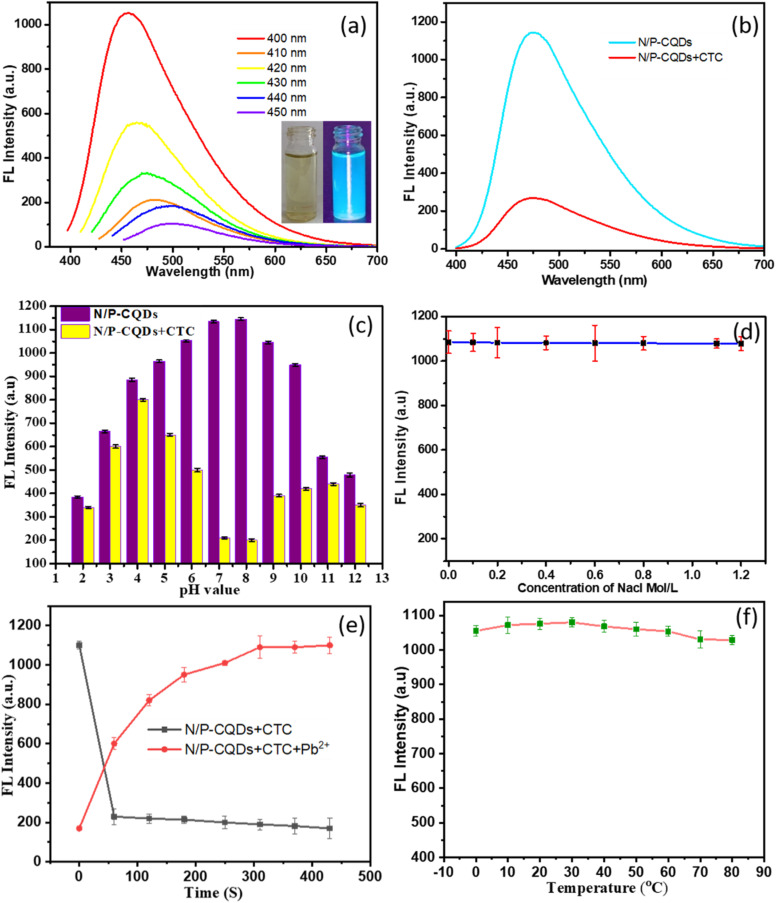
Study investigating the stability of N/P-CQDs by examining three variables: (a) excitation-dependent PL behaviors of N/P-CQDs and (b) fluorescence analysis of N/P-CQDs with and without CTC. (c) pH-dependent behavior of N/P-CQDs analysed in the pH range of 2 to 12. (d) Saline environment stability of N/P-CQDs analysed in different sodium chloride (NaCl) concentrations (0.1 to 1.2 mol L^−1^) to explore the change in their emission intensity. (e) Time-dependent fluorescence response of N/P-CQDs to CTC and Pb^2+^ ions. (f) Thermostability of N/P-CQDs analysed in the temperature range of 0 °C to 80 °C.

The efficacy and practical application of carbon quantum dots (N/P-CQDs) are greatly dependent on their stability. Detailed investigations into the photoluminescence (PL) properties of N/P-CQDs under various conditions reveal compelling insights, as summarized below. Investigation of their emission spectra involved recording them across a range of excitation wavelengths from 400 to 450 nm at 10 nm increments to explore the optical properties of the carbon quantum dots (N/P-CQDs). Fig. 6a depicts the PL behaviors of the CQDs, revealing a consistent excitation-dependent pattern. Notably, the strongest PL emission peak shifts towards a longer wavelength as the excitation wavelength increases, accompanied by a gradual decrease in PL intensity. These uniform trends across a range of excitation wavelengths for the N/P-CQDs suggest a systematic phenomenon intrinsic to the material rather than being sample-specific. The observed excitation-dependent PL behavior is likely influenced by factors such as the optical selection of differently-sized nanoparticles and the presence of distinct surface emission traps within the CQDs.

The feasibility of employing N/P-CQDs for the detection of (CTC) was confirmed by adding 80 μM CTC. The findings displayed in Fig. 6b reveal that the intrinsic fluorescence of the probe is rapidly quenched by CTC, taking place in less than 60 s, and the signal remains steady for 75 min. To improve the pH values for the sensor system, different pH Tris–HCl buffers were tested. According to the results, as seen in Fig. 6c, a pH of 8.0 yields the maximum fluorescence intensity ratio in the presence of CTC. By optimizing the pH, N/P-CQDs may detect CTC more effectively, highlighting the significance of careful parameter adjustment for the best possible sensor performance.

Fig. 6d shows that there is very little variation in the PL intensity of N/P-CQDs, even when the concentration of NaCl is increased to a high level of 0.1 to 1.2 mol L^−1^. This demonstrates their applicability in biological samples or complicated environments possessing a high ionic strength. Their salt tolerance is due to their abundant surface functional groups such as –COOH, –OH, and –NH_2_ and phosphate moieties, which confer significant colloidal stability *via* electrostatic repulsion and steric hindrance. These functions facilitate the dispersion and inhibit the aggregation of the particles under elevated ionic strength, hence safeguarding the emissive characteristics of the carbon quantum dots. The above-mentioned optimization strategy revealed the outstanding stability of N/P-CQDs in the presence of high salt concentrations. Fig. 6e shows a time-dependent response of N/P-CQDs to CTC and Pb^2+^ in terms of fluorescence. Upon the addition of CTC, the fluorescence intensity of N/P-CQDs was quickly quenched over 50 s and this result showed that CTC rapidly interacted with the CQDs. Alternatively, with the addition of Pb^2+^, their fluorescence increased steadily, proving that the time-dependent process of recovery was linked to the formation of the Pb^2+^–CTC complex. Fig. 6f shows that the PL intensity of N/P-CQDs did not decrease when the temperature increased from 10 °C to 80 °C. Ensuring a constant performance under various operating situations is contingent on the temperature stability of the probe. The substantial stability of N/P-CQDs is highlighted by these thorough results, which is crucial for their real-world use. N/P-CQDs are more adaptable and reliable for a wider range of applications because of their capacity to retain constant PL characteristics under a variety of environmental conditions. The observed changes in fluorescence intensity with pH and temperature were found to be reversible, given that the original fluorescence was restored upon returning to neutral pH or room temperature. This confirms the structural stability and reusability of the N/P-CQDs under varying environmental conditions.

### 3.3. Sensitivity analysis of N/P-CQDs for CTC detection

**Fig. 7:**
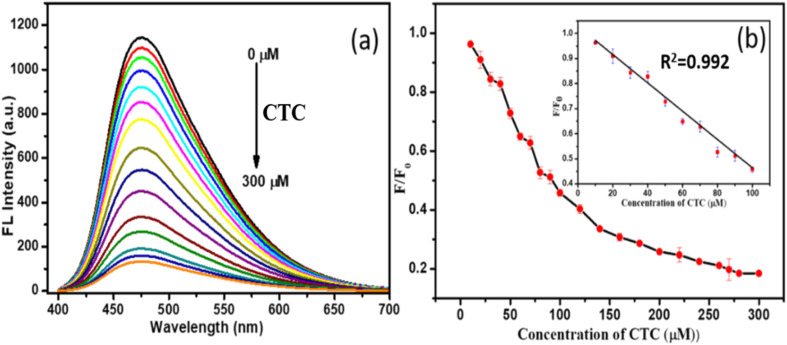
(a) Fluorescence emission spectra of N/P-CQDs (0.1 mL and 2 mg mL^−1^) in 1.5 mL Tris–HCl buffer (10 mM, pH 8.0) with an increasing concentration of chlortetracycline (CTC) ranging from 0 to 300 μM. (b) Stern–Volmer plot, with the inset illustrating linearity for CTC concentrations ranging from 10 to 100 μM.

The sensitivity of N/P-CQDs was evaluated by observing their fluorescence response to varying concentrations of chlortetracycline (CTC) from 0 to 300 μM (Fig. 7a). This assessment employed the excitation of N/P-CQDs under the optimal experimental conditions. The results demonstrate that the presence of CTC is associated with a steady reduction in the fluorescence of N/P-CQDs. Their response as a function of concentration (Fig. 7b) demonstrates the proportional relationship between CTC concentration and relative fluorescence intensity (*F*/*F*_0_) at an emission wavelength of 475 nm in the concentration range of 0.5 to 100 μM. The coefficient of determination is *R*^2^ = 0.992, signifying a high value.

The limit of detection (LOD) for CTC was established to be 30 nM with a signal-to-noise ratio of three, indicating the high sensitivity of N/P-CQDs in the detection of CTC. The comparison of the performance of N/P-CQDs for CTC detection with prior studies reveals either a reduced detection limit or an expanded linear range (Table 1). The findings indicate that N/P-CQDs possess a low limit of detection, a broad dynamic range, and high sensitivity for the detection of CTC. Also, the high sensitivity of N/P-CQDs make them appropriate for a range of analytical applications in environmental monitoring.

**Table 1:** Fluorescent sensors for the detection of CTC and Pb^2+^

Materials	Target	Working range	LOD	Ref.
N,S co-doped CDs	TCs	0.1–65 μM	0.04 μM	29
TP-CQD	CTC	0.5 to 60 μM	0.025 μM	30
CDs	CTC	0.8–10 μM	0.0169 μM	31
(N-CDs@MIPs)	CTC	6.67–111.33 μM	3.19 μM	32
(N-CDs)	TC	0.4–100.0 μM	0.0419 μM	33
CdTe QDs@ZIF-8	CTC	1–30 μM	0.037 μM	34
(CDs)	CTC	0.2–8.0 μM	0.03445 μM	35
(N-CQDs)	TC	3.32–32.26 μM	0.2791 μM	36
CDs	Pb^2+^	0–20 μM	0.11 nM	37
CDs	Pb^2+^	0.01–1.0 μM	0.59 nM	38
CDs	Pb^2+^	0–6 mM	5.05 μM	39
S-CQDs	Pb^2+^	0–12 μM	13.3 nM	40
CDs	Pb^2+^	30–130 μM	7.15 × 10^−4^ M	41
N/P-CQDs	Pb^2+^	5–80 μM	18 nM	Present work
N/P-CQDs	CTC	0–300 μM	0.030 μM	Present work

**Fig. 8:**
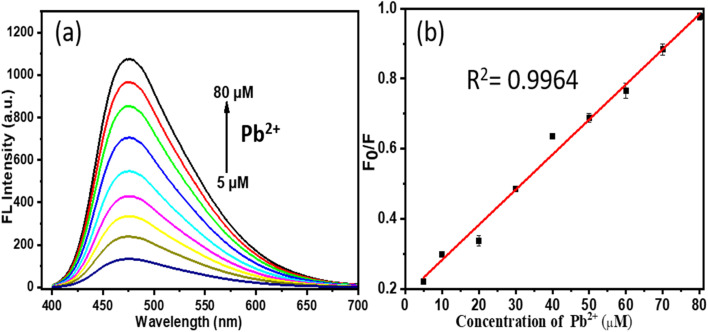
(a) Emission response of N/P-CQDs to different molar equivalents of Pb^2+^ (5 to 80 μM) in 1000 μL of Tris–HCl buffer (10 mM, pH = 8.0). (b) Stern–Volmer plot illustrating the linearity for Pb^2+^ concentrations in the range of 5 to 80 μM.

The sensitivity analysis for lead ions (Pb^2+^) reveals the effectiveness of the probe in sensing lead ions in water samples, as demonstrated by the subsequent analysis in the concentration range of 5 μM to 80 μM, as seen in Fig. 8a. Owing to the switching sensing mechanism of N/P-CQDs, their fluorescence is initially quenched by chlortetracycline (CTC), and then recovered with the introduction of Pb^2+^ ions. This restoration is attributed to the formation of a stable Pb^2+^–CTC complex, which reduces the interaction between CTC and the CQDs, facilitating the recovery of their fluorescence. The fluorescence intensity exhibits a linear correlation with Pb^2+^ concentration in this range, facilitating accurate quantification. This system demonstrates high sensitivity with a detection limit of 18 nM, enabling the effective detection of trace lead ion concentrations.

The limit of detection (LOD) for both CTC and Pb^2+^ was determined using the conventional equation, LOD = 3*σ*/*S*, where *σ* represents the standard deviation of the blank measurements (*n* = 10), and S is the slope of the linear calibration curve obtained from the fluorescence response, as seen in Fig. 8b. In the case of CTC, the LOD was calculated to be 30 nM based on the emission intensity at 475 nm. Similarly, the LOD for Pb^2+^ was found to be 18 nM. These values confirm the high sensitivity of N/P-CQDs and support their practical applicability in detecting trace levels of antibiotics and heavy metals in complex matrices.

### 3.4. Selectivity analysis

**Fig. 9:**
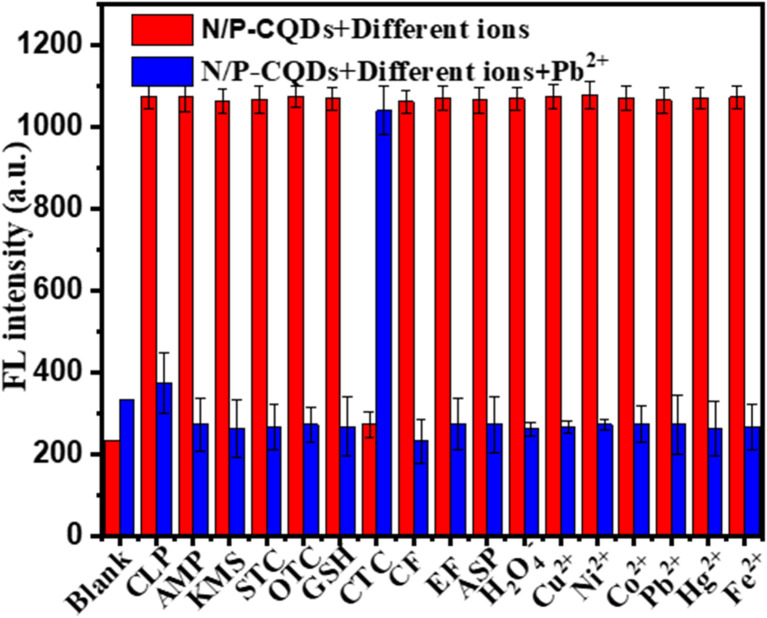
Emission response of N/P-CQDs to 100 μM of different ions including OTC, TC, STC, AMP, KMS, CLP, enrofloxacin (EF), ciprofloxacin (CF), and Cu^2+^, Ni^2+^, Hg^2+^, and Co^2+^ (red bar) and quenching of chlortetracycline (Pb^2+^: 80 μM) in the presence of various ions (100 μM) depicted by the blue bars.

We evaluated the selectivity of N/P-CQDs for chlortetracycline (CTC) through experiments involving structurally analogous antibiotics and amino acids typically present in environmental samples. The emission specificity of N/P-CQDs was thoroughly assessed through a detailed interference study to evaluate their performance in the presence of various potential interferents. The experimental strategy incorporated various interfering substances in place of CTC, specifically ions such as OTC, TC, STC, AMP, KMS, CLP, enrofloxacin (EF), ciprofloxacin (CF), anions, as well as cations including Cu^2+^, Ni^2+^, Hg^2+^, and Co^2+^. The concentration of both co-existing 100 μM and CTC was 80 μM, as indicated by the red bar in Fig. 9. In the competitive recovery experiments, Pb^2+^ was added following the introduction of interfering substances to assess the interference capabilities of the N/P-CQDs, as illustrated by the blue bar in Fig. 9. The concentration of CTC was set at 80 μM, while the volume of the other interfering substances was held constant at 100 μM.

This experimental setup facilitated a systematic evaluation of the capacity of N/P-CQDs to selectively identify CTC in the presence of various interfering substances. The red bar illustrates the emission response of the N/P-CQDs to various interferences, offering insights into the potential challenges presented by different ions. The introduction of Pb^2+^, as indicated by the blue bar, demonstrates the capacity to recover the fluorescence of N/P-CQDs. This study provides important insights into the reliability of the emission of N/P-CQDs a as sensor for the detection of CTC and Pb^2+^ ions in complex sample matrices. The fluorescence intensity of N/P-CQDs was observed upon exposure to various antibiotics and metal ions (100 μM each). The red bars represent quenching by different analytes and blue bars represent fluorescence recovery upon the addition of Pb^2+^ (80 μM) after quenching by CTC (80 μM). The results confirm the high selectivity of N/P-CQDs for the detection of CTC and Pb^2+^.

**Fig. 10:**
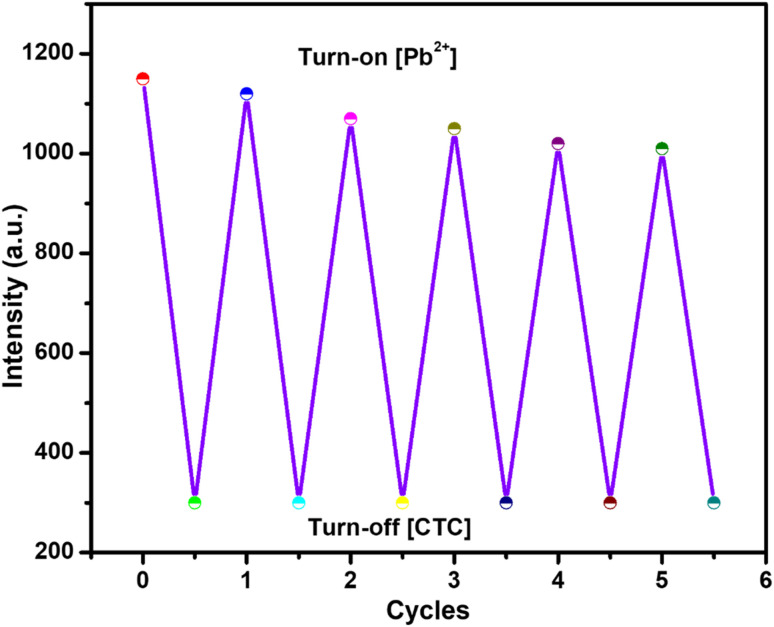
Reversible “on–off–on” fluorescence cycles of the N/P-CQD sensor, showing repeatable quenching by CTC and recovery by Pb^2+^, confirming its good reusability and stability.

The N/P-CQD-based dual-sensing probe was evaluated with respect to its reversibility and reusability in a sequential on–off–on fluorescence switching experiment using chlortetracycline (CTC) and Pb^2+^ ions. N/P-CQDs initially exhibited fluorescence emission at 475 nm, which was quenched when 30 μM CTC was added due to the inner filter effect, which corresponds to the off state. The fluorescence recovery (on state) was then introduced because of the addition of an equimolar concentration of Pb^2+^ (30 μM) due to the formation of a stable complex between CTC and Pb^2+^, which decreased the interaction between CTC and N/P-CQDs. This quenching and recovery behavior was effectively repeated five times in a series, as shown in Fig. 10, with a constant emission level and a signal loss of less than 5% following the 5th cycle. The findings support the conclusion that the N/P-CQDs show a high level of photostability, reversibility, and reuse to verify their reliability as a sensor in the repetitive detection of antibiotics/heavy metal ions in environmental samples.

### 3.5. Mechanism of chlortetracycline (CTC) detection

**Fig. 11:**
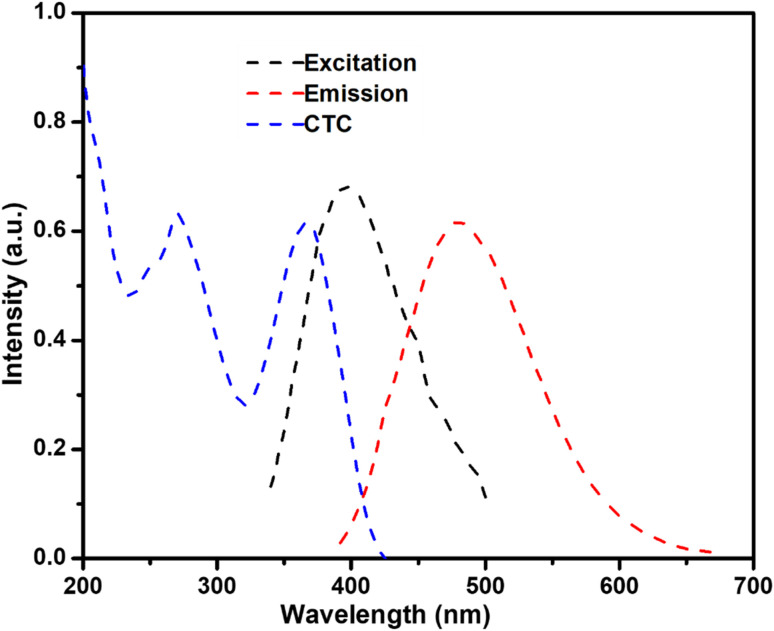
Spectral overlap of CTC absorption (blue) and N/P-CQD excitation (black, 400 nm) and emission (red, 475 nm).

The –NH_2_ (amine) and phosphorus-containing (P–O and P–N) groups facilitate the selective identification and coordination of CTC *via* hydrogen bonding, electrostatic interactions, and possible chelation. The surface functions enhance the efficient adsorption of CTC onto the CQD surface, disrupting the local optical environment and resulting in fluorescence quenching mainly through the inner filter effect (IFE). The spectral overlap features of the N/P-CQDs were further investigated to determine if their fluorescence quenching by CTC is attributed to the inner filter effect (IFE) mechanism. Fig. 11 indicates that the UV-Vis absorption spectrum of CTC has a wide absorption band in the range of 330–440 nm, which substantially overlaps with the excitation spectrum of N/P-CQDs at 400 nm. This overlap implies that the CTC molecules have the ability to absorb the excitation light that is to be absorbed by the N/P-CQDs, thus lowering the excitation energy reaching the carbon dots and reducing their fluorescence intensity. Conversely, the emission peak of the N/P-CQDs is observed at 475 nm, where the CTC absorption is much lower, indicating that the fluorescence decay is mainly caused by the primary inner filter effect (excitation-side quenching) and not reabsorption of the emitted light. These findings are a direct spectroscopic manifestation of the fact that the observed quenching is due to the inner filter effect and not charge transfer or nonpolar interactions. The introduction of Pb^2+^ ions leads to significant complexation with CTC, which decreases the absorbance of CTC, mitigates the IFE, and results in the recovery of the fluorescence of the N/P-CQDs. The above-mentioned functional groups are essential for the selective and sensitive on–off–on fluorescence sensing method for the simultaneous detection of CTC and Pb^2+^ ions.^42^

The analysis of the fluorescence quenching curve elucidated the underlying mechanism for the detection of CTC by the nitrogen/phosphorus-doped carbon quantum dots (N/P-CQDs). The fluorescence quenching curves were analysed in the presence and absence of CTC, utilizing CTC as an assay. A fluorescence lifetime analysis was conducted to determine the type of quenching induced by CTC. The addition of CTC did not influence the fluorescence lifespan, indicating that static quenching occurred instead of dynamic quenching. The results are presented in Fig. 3f. Additional research on the process of static quenching was conducted by timing the excitation spectra of the fluorescent probe. Consequently, the CTC absorbed the excitation light emitted by the probe, leading to a reduction in its fluorescence intensity, while maintaining its fluorescence lifespan (Fig. 8a). The results indicate that the mechanism responsible for the quenching of the fluorescence between N/P-CQDs and CTC is attributed to an inner filter effect (IFE). The restoration of fluorescence following the introduction of Pb^2+^ ions was ascribed to the robust interaction between Pb^2+^ and the CTC molecules. This interaction significantly mitigated the inner filter effect induced by CTC, thereby restoring the fluorescence signal of the N/P-CQDs. This dual sensing platform demonstrated linear responses for CTC (0–100 μM) with a detection limit of 30 nM and for Pb^2+^ ions (5–100 μM), highlighting its effectiveness in sample analyses.

### 3.6. Detection of CTC in real samples

Various real-world samples, such as tomato, milk, and river water, were utilized to examine the potential applications of this sensor. To assess the feasibility of the analytical strategy, the presence of CTC residues in the samples was evaluated using the conventional addition technique. Three distinct doses of CTC were subsequently introduced into each sample, *i.e.*, 20 μM, 30 μM, and 40 μM. The results presented in Table 2 indicate that the standard samples exhibit an average recovery rate of 96%. The repeatability (RSD%) value was below 1.92% (*n* = 3), with results of 96%, 99.8% and 99.9%, respectively. The findings offer additional support for the ability of N/P-CQDs to accurately detect CTC in real samples, demonstrating minimal interference from the matrix. Thus, N/P-CQDs offer an effective and reliable platform for monitoring CTC, presenting significant potential in food safety detection technology.

**Table 2:** Detection of chlortetracycline (CTC) in environmental samples

Sample	Spiked [CTC]/(μM)	Found CTC/(μM)	Recovery (%)	RSD (%)
Tomato	20	19.92	96	1.34
River water	30	29.94	99.8	1.28
Milk	40	39.98	99.9	1.92

To further confirm the ability of the N/P-CQD probe as a dual-sensing platform, real-sample recovery experiments for Pb^2+^ ions in tomato extract, river water and milk samples were performed under the same experimental conditions applied to the CTC detection. The respective samples were spiked with known concentrations of Pb^2+^ (20, 30 and 40 U L^−1^) and the fluorescence recovery response was measured. The recoveries were 92.0%, 96.7% and 94.3% with RSD values of less than 2.5%, as demonstrated in Table 3, in tomato, river water and milk, respectively. These findings support the fact that the fluorescence recovery can be attributed to the interaction between Pb^2+^ and CTC, which decreases the quenching ability of CTC on N/P-CQDs. The large recovery percentages and small RSD values prove its high accuracy, reproducibility and reliability in detecting Pb^2+^ ions in complex food and environment samples.

**Table 3:** Recovery analysis for Pb^2+^ in real samples using the N/P-CQDs dual-sensing platform (*n* = 3)

Sample	Spiked [Pb^2+^]/(μM)	Found Pb^2+^/(μM)	Recovery (%)	RSD (%)
Tomato	20	18.14	92	2.4
River water	30	29.02	96.7	1.8
Milk	40	37.72	94.3	2.2

## 4. Conclusion

In conclusion, an economical and eco-friendly approach was developed for synthesizing nitrogen and phosphorus-co-doped carbon quantum dots (N/P-CQDs) utilizing pomegranate peel juice as the carbon source. The synthesized N/P-CQDs exhibited superior fluorescence characteristics, with excitation and emission maxima of 400 nm and 475 nm, respectively, and a particle size of 7.5 nm, indicating their suitability for fluorescence sensing applications. This study presents a dual-target sensing system that successfully detects chlortetracycline (CTC) and lead (Pb^2+^) ions *via* an “on–off–on” fluorescence mechanism. Fluorescence quenching by CTC occurred through the inner filter effect (IFE), while the subsequent restoration of fluorescence in the presence of Pb^2+^ ions demonstrated the selectivity and sensitivity of the system. The developed sensor demonstrated a linear response for CTC (0–100 μM and LOD: 30 nM) and Pb^2+^ ions (5–100 μM), effectively detecting these analytes in real water samples with an LOD of 18 nM. The superior performance of N/P-CQDs in detecting both CTC and Pb^2+^ ions highlight their potential as an effective and adaptable platform for environmental monitoring and food safety applications. This study examines the shortcomings of conventional detection methods, presenting a streamlined synthesis pathway that lowers costs and minimizes environmental effects. However, broader validation across complex matrices, evaluation of its long-term stability and reusability, and further mechanistic studies are needed to advance this platform toward real-world applications. Surface modification of N/P-CQDs for enhanced selectivity, development of portable or integrated sensing platforms, exploration of multiplexed detection strategies, and evaluation of the sensor reusability were presented. The findings will facilitate additional investigation into biomass-derived CQDs for the detection of various pollutants, thereby advancing sustainable approaches in sensor development and environmental remediation.

## Author contributions

S. Viji, L. Srimathi Priya, A. Dinesh, K. Radhakrishnan, and Manikandan Ayyar: synthesized and characterized the samples, writing, graphical, and software work, construction plan, writing – original draft preparation, visualization, format checking. Prabhu Paramasivam, Lalitha Gnanasekaran, V. Mohanavel, and M. Santhamoorthy: rewriting, reviewing the manuscript and format checking, revision of the paper and discussion. G. Ramachandran, S. Santhoshkumar, Ankush Mehta, and N. Subasree: reviewing the manuscript and format checking, revision of the paper and discussion.

## Conflicts of interest

We declare that we have no known competing financial interests or personal relationships that could have appeared to influence the work reported in this paper.

## Data Availability

The data supporting the findings of this study are available from the corresponding author upon reasonable request.
